# Supplementary material for: Loss of tolerance precedes triggering and lifelong persistence of pathogenic type I interferon autoantibodies
Source: J Exp Med. 2024 Jul 17;221(9):e20240365. doi: 10.1084/jem.20240365 (PMC11253716; doi:10.1084/jem.20240365)
Supplement: Table S1 — shows baseline patient characteristics of the study sub-cohorts. [file JEM_20240365_TableS1.docx]

**Table S1. Baseline patient characteristics of the study sub-cohorts**

| **Characteristic** | **Patients >65 yrs with**  **anti-IFN-I autoAbs**  **(n = 35)** | **Patients >65 yrs without**  **anti-IFN-I autoAbs**  **(n = 1841)** | **Patients treated with pegylated IFNα**  **(n = 300)** |
| --- | --- | --- | --- |
|  | **n (%) or median (interquartile range)** | | |
| Year of birth | 1950 (1945-1953) | 1949 (1943-1954) | 1965 (1960-1970) |
| Female | 2 (5.7) | 334 (18.1) | 54 (18.1) |
| Ethnicity (white) | 31 (88.6) | 1704 (92.6) | 281 (94.0) |
| Ethnicity (black) | 3 (8.6) | 84 (4.6) | 5 (1.7) |
| Ethnicity (hispanic) | 1 (2.9) | 16 (0.9) | 3 (1.0) |
| Ethnicity (asian) | 0 (0.0) | 28 (1.5) | 8 (2.7) |
| Ethnicity (other/unknown) | 0 (0.0) | 9 (0.5) | 2 (0.7) |
| Risk group^#^ (HET) | 8 (22.9) | 778 (42.3) | 19 (6.3) |
| Risk group^#^ (IDU) | 3 (8.6) | 73 (4.0) | 164 (54.7) |
| Risk group^#^ (MSM) | 22 (62.9) | 875 (47.5) | 105 (35.0) |
| Risk group^#^ (other/unknown) | 2 (5.7) | 115 (6.2) | 12 (4.0) |
| CD4 (baseline, cells/mm^3^) | 303 (156-525) | 295 (130-487) | 384 (210-572) |
| HIV-1 RNA (baseline, log_10_) | 4.77 (4.07-5.12) | 4.69 (3.81-5.28) | 4.24 (3.47-5.09) |

Abbreviations: IFN-I = type I interferon; autoAbs = autoantibodies; HET = heterosexual; IDU = intravenous drug use; MSM = men who have sex with men.

^#^Risk group refers to most likely source of HIV-1 infection.
