# Supplementary material for: Loss of tolerance precedes triggering and lifelong persistence of pathogenic type I interferon autoantibodies
Source: J Exp Med. 2024 Jul 17;221(9):e20240365. doi: 10.1084/jem.20240365 (PMC11253716; doi:10.1084/jem.20240365)
Supplement: Table S2 — shows the impact of neutralizing anti-IFNα autoAbs on recorded outcomes. [file JEM_20240365_TableS2.docx]

**Table S2. Impact of neutralizing anti-IFNα autoAbs on recorded outcomes**

| **Outcome** | **Patients with**  **neutralizing anti-IFNα autoAbs**  **(n = 16)** | **Patients without**  **neutralizing anti-IFNα autoAbs**  **(n = 62)** | ***P* value** |
| --- | --- | --- | --- |
|  | **n (%) or median (interquartile range)** | |  |
| CD4 count (cells/mm^3^) | 612 (400-819) | 548 (395-663) | 0.319^#^ |
| CD8 count (cells/mm^3^) | 530 (331-1079) | 693 (459-1047) | 0.319^#^ |
| Aspergillosis | 0 (0.0) | 1 (1.6) | 1* |
| Bacterial pneumonia | 3 (18.8) | 7 (11.3) | 0.707* |
| Candidiasis (esophageal) | 1 (6.3) | 1 (1.6) | 0.874* |
| Candidiasis (oral) | 0 (0.0) | 2 (3.2) | 1* |
| **COVID-19 (severe, hospitalized)^$^** | **3 (18.8)** | **1 (1.6)** | **0.033*** |
| Diabetes | 1 (6.3) | 6 (9.7) | 1* |
| Encephalopathy (HIV-related) | 0 (0.0) | 2 (3.2) | 1* |
| Herpes simplex (mucocutaneous) | 1 (6.3) | 0 (0.0) | 0.462* |
| Herpes zoster | 0 (0.0) | 5 (8.1) | 0.547* |
| HIV-1 (log_10_ RNA) | 0.00 (0.00-0.00) | 0.00 (0.00-0.00) | 0.949^#^ |
| Mycobacterium avium (disseminated) | 0 (0.0) | 1 (1.6) | 1* |
| Neoplasms | 2 (12.5) | 18 (29.0) | 0.303* |
| Non-Hodgkin’s lymphoma | 0 (0.0) | 2 (3.2) | 1* |
| Pneumocystis pneumonia | 0 (0.0) | 2 (3.2) | 1* |

Abbreviations: IFN-I = type I interferon; autoAbs = autoantibodies.

^#^Wilcoxon rank-sum test

*Fisher’s exact test

^$^For percentage calculations shown here, the indicated total n was used. In the Fig. 3 calculations, n differs as only patients who were still actively enrolled in the SHCS were included
