# Supplementary material for: Loss of tolerance precedes triggering and lifelong persistence of pathogenic type I interferon autoantibodies
Source: J Exp Med. 2024 Jul 17;221(9):e20240365. doi: 10.1084/jem.20240365 (PMC11253716; doi:10.1084/jem.20240365)
Supplement: Table S3 — shows the impact of prior events on development of anti-IFN-I autoAbs. [file JEM_20240365_TableS3.docx]

**Table S3. Impact of prior events on development of anti-IFN-I autoAbs**

| **Prior event** | **Patients who developed anti-IFN-I autoAbs**  **(n = 35)** | **Patients who did not develop anti-IFN-I autoAbs**  **(n = 138)** | ***P* value** |
| --- | --- | --- | --- |
|  | **n (%) or median (interquartile range)** | |  |
| Years since HIV diagnosis | 14.57 (7.69-23.15) | 14.65 (6.47-20.33) | 0.327^#^ |
| CD4 count (cells/mm^3^) | 510 (354-751) | 481 (319-720) | 0.479^#^ |
| CD4 count, nadir (cells/mm^3^) | 130 (62-195) | 147 (68-225) | 0.395^#^ |
| CD8 count (cells/mm^3^) | 782 (540-1123) | 812 (553-1131) | 0.880^#^ |
| Bacterial pneumonia | 0 (0.0) | 7 (5.1) | 0.379* |
| Candidiasis (esophageal) | 3 (8.6) | 7 (5.1) | 0.699* |
| Candidiasis (oral) | 9 (25.7) | 34 (24.6) | 1* |
| Candidiasis (vulvovaginal) | 1 (2.9) | 1 (0.7) | 0.866* |
| Cryptococcosis (disseminated) | 0 (0.0) | 1 (0.7) | 1* |
| Cryptosporidiosis (diarrhea > 1 mo) | 0 (0.0) | 1 (0.7) | 1* |
| **Cytomegalovirus (IgG positivity)^$^** | **25 (71.4)** | **120 (87.0)** | **0.015*** |
| Cytomegalovirus (retinitis) | 0 (0.0) | 2 (1.4) | 1* |
| Diabetes | 1 (2.9) | 16 (11.6) | 0.218* |
| Encephalopathy (HIV-related) | 1 (2.9) | 2 (1.4) | 1* |
| Hepatitis B virus (IgG positivity) | 25 (71.4) | 79 (57.2) | 0.194* |
| Hepatitis C virus (IgG positivity) | 2 (5.7) | 9 (6.5) | 1* |
| Herpes simplex (mucocutaneous) | 1 (2.9) | 0 (0.0) | 0.457* |
| **Herpes zoster^$^** | **12 (34.3)** | **21 (15.2)** | **0.020*** |
| HIV-1 (log_10_ RNA) | 0.00 (0.00-1.64) | 0.00 (0.00-0.00) | 0.199^#^ |
| Kaposi’s sarcoma | 1 (2.9) | 10 (7.2) | 0.574* |
| Microsporidiosis | 0 (0.0) | 1 (0.7) | 1* |
| Mycobacterium avium (disseminated) | 0 (0.0) | 1 (0.7) | 1* |
| Myelopathy (HIV-related) | 0 (0.0) | 1 (0.7) | 1* |
| Neoplasms | 5 (14.3) | 10 (7.2) | 0.324* |
| Neuropathy (peripheral, HIV-related) | 2 (5.7) | 1 (0.7) | 0.195* |
| Non-Hodgkin’s lymphoma | 3 (8.6) | 2 (1.4) | 0.093* |
| Oral hairy leukoplakia | 8 (22.9) | 25 (18.1) | 0.692* |
| Pneumocystis pneumonia | 3 (8.6) | 12 (8.7) | 1* |
| Smoking (baseline, ever) | 15 (42.9) | 84 (60.9) | 0.067* |
| Syphilis (screening test) | 8 (22.9) | 24 (17.4) | 0.674* |
| Toxoplasmosis (screening test) | 18 (51.4) | 80 (58.0) | 0.445* |
| Thrombocytopenia (HIV-related) | 3 (8.6) | 6 (4.3) | 0.563* |
| Tuberculosis (latent) | 2 (5.7) | 5 (3.6) | 0.990* |
| Tuberculosis (pulmonary) | 2 (5.7) | 3 (2.2) | 0.581* |

Abbreviations: IFN-I = type I interferon; autoAbs = autoantibodies.

^#^Wilcoxon rank-sum test

*Fisher’s exact test

^$^For percentage calculations shown here, the indicated total n was used. In the Fig. 4 percentage calculations, n differs slightly as only patients with complete data for the indicated parameter were included
